# Ferroptosis-Related Transcriptional Level Changes and the Role of CIRBP in Glioblastoma Cells Ferroptosis

**DOI:** 10.3390/biomedicines13010041

**Published:** 2024-12-27

**Authors:** Zijiang Yang, Ting Zhang, Xuanlin Zhu, Xiaobiao Zhang

**Affiliations:** 1 Department of Neurosurgery, Zhongshan Hospital, Fudan University, Shanghai 200032, China; 22111210107@m.fudan.edu.cn; 2 Department of Central Laboratory, Jiangyin Clinical College of Xuzhou Medical University, Jiangyin 214400, China; yidatina@126.com; 3 School of Basic Medical Sciences, Naval Medical University (Second Military Medical University), Shanghai 200433, China; 2259028@tongji.edu.cn; 4 Cancer Center, Zhongshan Hospital, Fudan University, Shanghai 200032, China; 5 Digital Medical Research Center, Fudan University, Shanghai 200032, China

**Keywords:** ferroptosis, glioma progression, lipid peroxidation, RNA sequencing, cold-inducible RNA-binding protein, endoplasmic reticulum stress

## Abstract

Background/Objective: We aimed to elucidate the roles of ferroptosis-associated differentially expressed genes (DEGs) in glioblastoma and provide a comprehensive resource for researchers in the field of glioblastoma cell ferroptosis. Methods: We used RNA sequencing to identify the DEGs associated with erastin-induced ferroptosis in glioblastoma cells. We further unraveled the biological functions and clinical implications of cold-inducible RNA-binding protein (CIRBP) in the context of glioblastoma by using a multifaceted approach, encompassing gene expression profiling, survival analysis, and functional assays to elucidate its role in glioblastoma cell mortality and its potential influence on patient prognosis. Results: We identified and validated the gene encoding CIRBP, the expression of which is altered during glioblastoma ferroptosis. Our findings highlight the relationship between CIRBP expression and ferroptosis in glioblastoma cells. We demonstrated that CIRBP modulates key aspects of cell death, thereby altering the sensitivity of glioblastoma cells to erastin-induced ferroptosis. A prognostic model, constructed based on *CIRBP* expression levels, revealed an association between lower *CIRBP* levels and poorer prognosis in glioma patients; this finding was corroborated by our comprehensive in vitro and in vivo assays that highlighted the impact of modulating CIRBP expression on glioblastoma cell viability and ferroptotic response. Conclusion: Our research unravels the complex molecular dynamics of ferroptosis in glioblastoma and underscores CIRBP as a potential biomarker and therapeutic target. This improved understanding of the role of CIRBP in ferroptosis paves the way for more precise and efficacious treatments for glioblastoma, potentially improving patient outcomes.

## 1. Introduction

Glioblastoma, which arises from neuroepithelial cells and is characterized by diffuse growth within the brain parenchyma, is also defined as diffuse astrocytic glioma. It is molecularly distinguished by a lack of *Isocitrate Dehydrogenase* (*IDH*) and *histone H3* gene mutations, along with *Telomerase Reverse Transcriptase* (*TERT*) promoter mutation, *Epidermal Growth Factor Receptor* (*EGFR*) gene amplification, and a +7/−10 cytogenetic profile [1,2]. Glioblastoma (GBM) is molecularly distinct from other brain tumors due to several key features. Unlike lower-grade gliomas such as astrocytomas and oligodendrogliomas, GBM is *IDH* wild-type (IDHwt), meaning it lacks the *IDH* mutations commonly found in these tumors, which are associated with better prognosis and slower progression. GBM’s lack of histone H3 mutations (e.g., H3K27M) further distinguishes it from pediatric gliomas and midline gliomas, where such mutations are prevalent and linked to a more indolent clinical course. A hallmark of GBM is the presence of *TERT* promoter mutations, which are nearly ubiquitous in GBM and activate telomerase, promoting tumor cell immortality, whereas these mutations are less common in other gliomas like oligodendrogliomas. GBM is also characterized by *EGFR* amplification, particularly the *EGFRvIII* mutant, which is central to its aggressive growth and resistance to treatment—this is a defining feature of GBM and rarely seen in other tumors like meningiomas or low-grade astrocytomas. Additionally, GBM typically exhibits a +7/−10 cytogenetic profile (gain of chromosome 7 and loss of chromosome 10), whereas oligodendrogliomas show 1p/19q codeletion, another distinguishing genetic feature. These molecular differences set GBM apart from other gliomas and brain tumors, contributing to its aggressive nature and poor prognosis [3]. As the most prevalent intracranial malignant tumor, glioblastoma has a dismal prognosis, with patients typically experiencing relapse despite conventional therapy; the disease has a median survival time of 8–15 months and a five-year survival rate of only 6.8% [2,4,5]. Temozolomide, an alkylating agent, has become a standard treatment for glioblastoma (GBM) due to its antitumor activity, which is enhanced in GBM cells with deficient mismatch repair mechanisms, such as those with mutations in the *MGMT* gene (encoding O6-methylguanine-DNA methyltransferase), that cannot effectively repair the DNA damage caused by methylated adducts, which slightly improves the prognosis: the median survival time for patients with glioblastoma treated with temozolomide is approximately 14.6 months, with a 5-year survival rate of 9.8%; these figures rise to 23.4 months and 13.8% in patients with O^6^-methylguanine-DNA-methyltransferase (MGMT) promoter methylated glioblastoma [6].

In recent years, advancements in treatment technologies, including safer total resection, chemotherapy, radiotherapy, immune checkpoint blocking therapy, and immune cell therapy, have extended patient survival, although the outcomes remain far from optimal [7]. A wealth of high-quality glioma research has emerged, addressing the disease at the DNA [8], transcriptional [9], and cellular levels [10]. We have contributed to this body of work by identifying differentially expressed prognostic mRNAs, long non-coding RNAs (lncRNAs), and microRNAs (miRNAs) in gliomas and normal brains. For example, we constructed a lncRNA–miRNA–mRNA network to uncover the potential significance of *Prostate Androgen-Regulated Transcript 1* (*PART1*)-hsa-mir-25-*Talin 2* (*TLN2*)/*Zinc Finger DHHC-Type Palmitoyltransferase 8* (*ZDHHC8*) in glioma. Our findings also indicate dysregulation of mitochondrial transcription gene families and a disordered immune environment in glioma [11]. These insights provide a molecular basis for understanding glioma pathogenesis and development and underscore the need for more effective and specific therapeutic targets to enhance patient survival.

Ferroptosis is a tightly regulated, non-apoptotic form of cell death that is mechanistically distinct from apoptosis, necrosis, and autophagy, characterized predominantly by iron-dependent lipid peroxidation and aberrant oxidative stress. This process is initiated by the accumulation of reactive oxygen species (ROS) and is exacerbated by the depletion of cellular antioxidant defenses, particularly the glutathione (GSH)-dependent activity of glutathione peroxidase 4 (GPX4). Iron plays a pivotal role through its participation in Fenton chemistry, generating highly reactive hydroxyl radicals that catalyze lipid peroxidation. Additionally, the oxidation of polyunsaturated fatty acids (PUFAs) within membrane phospholipids further amplifies peroxidative damage, leading to the destabilization of cellular membranes and eventual cell death. These foundational mechanisms provide critical insights into the molecular pathways governing ferroptosis, particularly in the context of glioblastoma, and highlight its potential as a therapeutic target in this aggressive malignancy [12]. Researchers have increasingly recognized ferroptosis as a critical reaction in cancer cells following radiotherapy, chemotherapy, and immunotherapy, suggesting the potential of using ferroptosis inducers in combination with conventional treatments [13]. In the context of glioma, research has shown that mRNAs, lncRNAs, circRNAs, and microRNAs can regulate ferroptosis through pathways involving hydrogen peroxide, iron, and lipid remodeling. Notably, Activating Transcription Factor 3 (ATF3) enhances glioma cell ferroptosis by promoting hydrogen peroxide and iron accumulation [14], whereas Activating Transcription Factor 4 (ATF4) triggers ferroptosis through Solute Carrier Family 7 Member 11 (SCL7A11)/SCX-dependent mechanism [15]. In addition, MDM2 Proto-Oncogene (MDM2) and MDM4 Regulator Of P53 (MDMX) regulate Peroxisome Proliferator-Activated Receptor Alpha (PPARα)-mediated lipid remodeling, leading to glioma cell ferroptosis [16]. Overexpression of RAS-selective lethal 3 (RSL3) induces ferroptosis through the nuclear factor kappa-B (NF-κB) pathway in glioblastoma [17], whereas microRNA-670-3p inhibits Acyl-CoA Synthetase Long-Chain Family Member 4 (ACSL4) expression, suppressing glioblastoma cell ferroptosis [18]. Additionally, glutathione peroxidase 4 (GPX4) is an important negative regulator of ferroptosis, as it reduces intracellular lipid peroxides and protects cells from oxidative stress. In glioma, inhibition of GPX4 can accelerate the occurrence of ferroptosis. Therefore, the use of GPX4 inhibitors can effectively induce ferroptosis in glioma cells [19]. Another important regulatory factor is low-density lipoprotein receptor-related protein 1 (LRP1), which plays a key role in ferroptosis by regulating lipid and iron uptake in cells. In glioma, the upregulation of LRP1 expression is associated with increased sensitivity to ferroptosis [20]. The mitogen-activated protein kinase (MAPK) signaling pathway is also believed to be involved in the regulation of ferroptosis, especially through the activation of p38 MAPK, which can enhance the effects of ferroptosis. However, its role in glioma ferroptosis still requires further experimental validation [21,22,23].

Furthermore, resistance to temozolomide, immune cell infiltration, and checkpoint-targeted therapies are also linked to ferroptosis, with studies highlighting the roles of various lncRNAs and microRNAs in these processes [24,25,26].

In the present study, we aimed to filter and analyze the differentially expressed mRNAs and lncRNAs in glioma ferroptosis to further understand its pathophysiological mechanisms and identify effective biomarkers. We investigated transcriptional changes after glioma cell ferroptosis, dissected the function and potential regulatory relationships among differentially expressed mRNAs and lncRNAs, and explored whether cold-inducible RNA-binding protein (CIRBP) promotes glioma cell ferroptosis. Additionally, we aimed to enhance the understanding of the mRNAs and lncRNAs associated with glioma ferroptosis, which we expect would influence the development of potential ferroptosis-related glioma therapies.

## 2. Materials and Methods

### 2.1. Cell Culture and Sample Preparation

Glioma U87 cell and A172 cell lines were obtained from The Cell Bank of Type Culture Collection of Chinese Academy of Sciences (Shanghai, China) and cultured in Dulbecco’s Modified Eagle Medium (Thermo Fisher Scientific, Waltham, MA, USA) supplemented with 10% fetal bovine serum (Gibco, Thermo Fisher Scientific, Waltham, MA, USA), 100 IU/mL penicillin, and 100 µg/mL streptomycin (Invitrogen, Carlsbad, CA, USA), according to standard protocols [27]. Cells were incubated in a Therma incubator (Thermo Fisher Scientific) at 37 °C under a 5% CO_2_ atmosphere. The ferroptosis inducer erastin was purchased from MedChem Express (South Brunswick Township, NJ, USA). U87 and A172 cells treated with 20 μmol erastin were used for subsequent experiments, with U87 and A172 cells treated with 20 μmol dimethylsulfoxide serving as negative controls.

### 2.2. RNA Isolation and Quality Control

RNA samples of U87 treated with 20 μmol erastin and U87 treated with DMSO during 4 days were extracted using TRIzol reagent (Invitrogen), according to the manufacturer’s protocol. The purity and integrity of the collected RNA were evaluated using an Agilent 2100 TapeStation (Agilent Technologies, Santa Clara, CA, USA), and the RNA concentration was measured using a Qubit RNA Assay Kit and Qubit Fluorometer (Invitrogen). Samples with an RNA integrity number greater than 7.0 were selected for subsequent experiments.

### 2.3. cDNA Library Preparation and Computational Analysis Process

CapitalBio Technology (Beijing, China) sequenced and generated a sequencing library using Agilent 2100 and a KAPA Library Quantification kit (KAPA Biosystems, Cape Town, South Africa) to prepare the RNA-seq library. The final libraries were sequenced using an Illumina HiSeq sequencer (Illumina, San Diego, CA, USA). After obtaining raw RNA sequencing data, FastQC (Version: 0.11.9) was used to filter, count, and clean the data. Sequences were aligned using HISAT2 (Version: 2.2.1)/Tophat2 (Version: 2.1.1) and assembled with StringTie (Version: 2.2.1), and gene quantification was performed using featureCounts (Version: 2.0.1) and StringTie (Version: 2.2.1) software. The R package “limma” (Version: 3.62.1) was used to filter differentially expressed genes (DEGs) using the criteria: |log2FC| ≥ 1, *p*-value ≤ 0.05, with at least two-thirds of samples showing gene expression in case/control groups and a mean value ≥1.

### 2.4. Prediction of lncRNA Targets

We used three methods to predict lncRNA targets, as follows:

1. Complementary base pairing: Using lncTar [28], lncRNA–mRNA interactions were predicted via free energy minimization. Then, Fast All Sequences in A (FASTA) sequences of lncRNA and mRNA were used to calculate the normalized binding free energy (ndG), with ndG < –0.1 indicating potential interactions. These interactions were input into Cytoscape (Version: 3.9.0) to identify hub lncRNA–mRNA interaction models.
2. Cis- and trans-regulation prediction: LncRNA target predictions considered the distance between lncRNA and mRNA and their interaction with cis-acting elements (cis-regulation) or trans-acting factors (trans-regulation), which are the two main factors that distinguish the cis-regulation or trans-regulation of lncRNAs. Prediction of lncRNA cis-regulation target genes suggests that lncRNAs are located upstream and downstream of co-expressed genes that could intersect with promoters or other cis-acting elements, thereby regulating gene expression at the transcriptional or post-transcriptional level. LncRNA trans-regulation target gene prediction suggests that the function of lncRNA is not related to the position between lncRNA and the coding gene but is based on correlation analysis between lncRNA and protein-coding gene co-expression analysis. Co-expression analysis was performed using a Pearson correlation coefficient > 0.99 and a *p*-value < 0.05. Based on the co-expression results of the lncRNAs and mRNAs, the presence of cis-targeted regulation was used to determine the lncRNA target mRNAs.
3. Competing endogenous RNA (ceRNA) network: MiRNAs bind to targeted mRNAs and inhibit mRNAs expression, whereas lncRNAs can act as microRNA sponges, leading to microRNA downregulation and resulting in the upregulation of miRNA-targeted mRNAs. We constructed a ceRNA network based on lncRNA–mRNA co-expression analysis and predicted shared miRNAs using TargetScan, with miRNAs serving as bridges in the lncRNA–microRNA–mRNA ceRNA network.

### 2.5. LncRNA Transcription Factor (TF) Prediction

TFs are proteins with specialized structures that regulate gene expression by binding to gene regulatory regions. The JASPAR database (2022) and TFBS Tools (MEME Suite: 5.5.1, FIMO: 5.5.1, TESS: Web-based, RSAT: 2023, PSCAN: 2.0) were used to predict the binding sites, orientation, and scoring in the regions 2000 bp upstream and 500 bp downstream of the start site of each lncRNA.

### 2.6. Gene Ontology (GO), Pathway, and Disease Analyses

Using KOBAS, which contains seven pathway databases, five human disease databases, and one functional database, differentially expressed lncRNA-targeted mRNAs were selected for GO, pathway, and disease functional enrichment analyses to investigate the potential role of lncRNAs. We set a false discovery rate (FDR) < 0.05 as the threshold value.

### 2.7. Protein–Protein Interaction (PPI) Networks

PPI networks can be classified into two main types: physical and genetic. Physical interaction refers to the chemical reaction between proteins through spatial conformation or chemical bond binding, whereas genetic interaction means that the phenotypic changes in proteins/genes are affected by other proteins/genes under special circumstances. We used the STRING database, which integrates multiple data sources, to score and screen protein interactions based on a combined score. A combined score > 400 between two proteins or genes was extracted from the STRING database. Then, PPI pairs were loaded into Cytoscape to visualize the complex networks. CytoHubba (Version: 0.1.1) was used to calculate and screen important genes.

### 2.8. lncRNA–mRNA–RNA-Binding Protein (RBP) Prediction

RBPs have important regulatory roles in RNA metabolism processes, such as post-transcriptional splicing, modification, transport, translation, and degradation. Shared RBPs of lncRNAs and mRNAs were predicted using the ENCODE database based on the lncRNA–mRNA correlation results.

### 2.9. Cell Proliferation

Cells were treated with 20 µM erastin or 10 µM ferrostatin 1 and/or gene transfection. Transfected cells and negative control suspensions were seeded in 96-well plates at 2000 cells per well, and the treated cells were seeded in triplicate. Cells were treated with 20 µM erastin or 10 µM ferrostatin 1 or DMSO as negative control after 24 h of cells seeded. The culture plate was placed in an incubator for 24 h, and the cells were measured every 24 h. Subsequently, 10 μL of Cell Counting Kit-8 solution (MCE, Monmouth Junction, NJ, USA) was added 4 h before measurement, and the absorbance at 450 nm was determined using a microplate reader.

### 2.10. Cell Transfection

The siRNA for CIRBP knockdown, the pcDNA3.1-CIRBP overexpression vector, and the corresponding control were acquired from Genomeditech (Genomeditech, Shanghai, China). Liposome 3000 (Invitrogen, Carlsbad, CA, USA) was used to transfect the pcDNA3.1 vector into cells, according to the manufacturer’s instructions. Hieff Trans^®^ Liposomal Transfection Reagent (Yeasen, Shanghai, China) was used to transfect siRNA according to the manufacturer’s instructions. The sequences for CIRBP are listed: siRNA-1: CUUCUCAAAGUACGGACAGAU, siRNA-2: GCCAUGAAUGGGAAGUCUGUA, siRNA-NC: UUCUCCGAACGUGUCACGU.

### 2.11. Quantitative Reverse Transcription Polymerase Chain Reaction (qRT-PCR)

The RNA samples were extracted using TRIzol reagent (Invitrogen). After assessing the RNA quality and quantity, qualified RNA samples were reverse-transcribed to a cDNA library. qRT-PCR was conducted using the qPCR SYBR Green Master Mix (Thermo Fisher Scientific) and primers purchased from Shenggong (Shenggong, Shanghai, China). GAPDH was used as an endogenous control. The primers used were CIRBP forward: TTAGGAGGCTCGGGTCGTT, reverse: CATGGCGGCCACTGAGTC; GAPDH forward: GGAGCGAGATCCCTCCAAAAT, reverse: GGCTGTTGTCATACTTCTCATGG.

### 2.12. Western Blotting

Cell proteins were collected using RIPA buffer (Beyotime, Beijing, China) containing protease and phosphatase inhibitors. Equal amounts of protein were separated via SDS-PAGE before being transferred onto PVDF membranes. The membranes were blocked with 5% milk in tris-buffered saline and polysorbate 20 (TBST) and incubated overnight at 4 °C with the primary antibodies against CIRBP (Proteintech, Wuhan, China), glutathione peroxidase 4 (GPX4) (Abcam, Cambridge, MA, USA), and SLC7A11 (Abcam, Cambridge, MA, USA). We used GAPDH (Abcam, Cambridge, MA, USA) as the control. After three washes with TBST, membranes were incubated with secondary antibodies (Abcam, Cambridge, MA, USA). Finally, the proteins were visualized using a Bio-Rad ChemiDoc MP (Bio-Rad Laboratories, Hercules, CA, USA).

### 2.13. Statistical Analysis

Special analysis methods and cut-off values are described in Section 2.2, Section 2.3, Section 2.4, Section 2.5, Section 2.6, Section 2.7. Quantitative data are presented as mean ± standard deviation. A paired Student’s *t*-test was performed using GraphPad Prism 8. All experiments were repeated at least three times. The significance threshold was set at *p* < 0.05.

## 3. Results

### 3.1. Erastin-Induced Glioma Cell Ferroptosis

**Figure 1:**
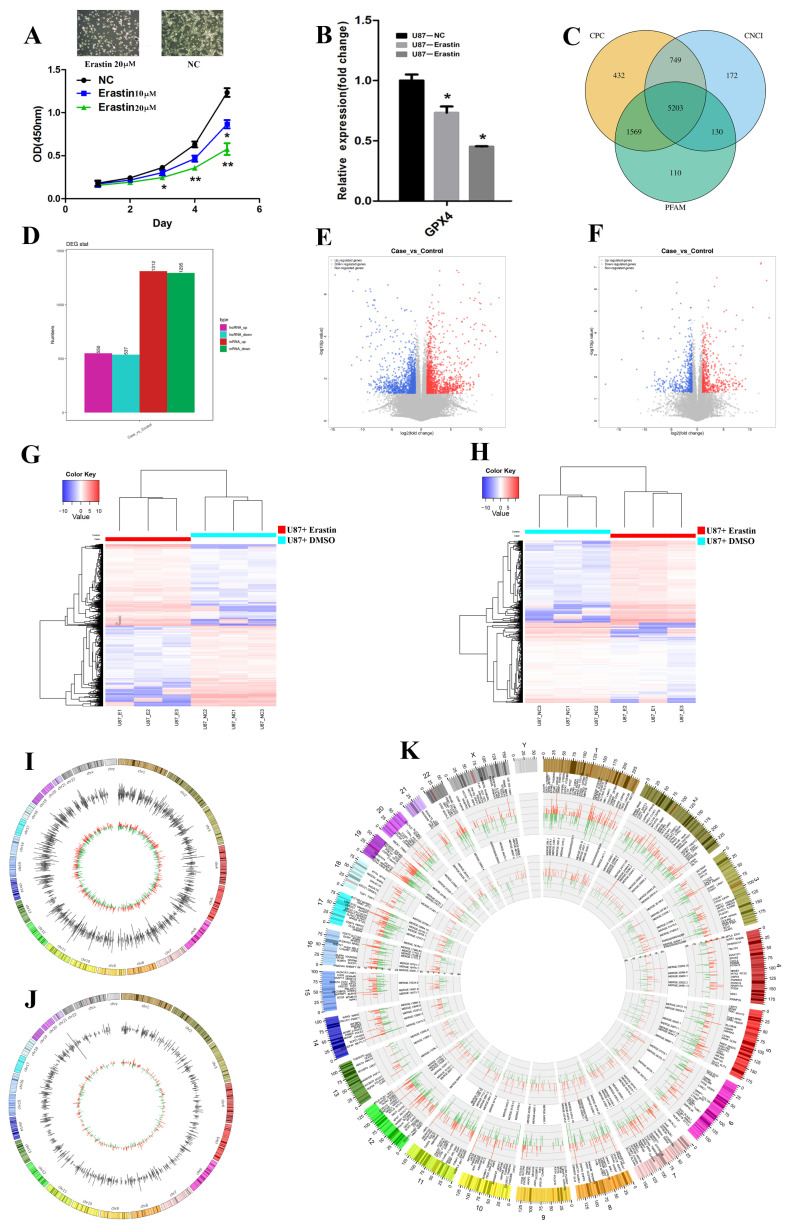
Transcriptional changes induced by erastin in glioma cells post-ferroptosis: (**A**) Effectiveness of erastin at concentrations of 10 and 20 µM in inducing ferroptosis; 20 µM erastin showed a significant increase in the degree of ferroptosis induced compared with that for the control. (**B**) Reduced expression of the ferroptosis marker gene *GPX4* after erastin treatment, confirming ferroptosis. (**C**) Identification of novel lncRNAs with no coding capacity as predicted by CPC (Coding Potential Calculator), CNCI (Coding–Non-Coding Index), and PFAM (Protein Families Database) tools. (**D**) Significantly upregulated and downregulated mRNAs and lncRNAs in ferroptosis-induced glioma cells compared to controls. (**E**) Volcano plot of differentially expressed mRNAs. (**F**) Volcano plot of differentially expressed lncRNAs. (**G**) Heatmap of mRNA expression patterns, distinguishing ferroptosis samples from controls. (**H**) Heatmap of lncRNA expression patterns, distinguishing ferroptosis samples from controls. (**I**) Distribution of dysregulated mRNAs across chromosomes. (**J**) Distribution of dysregulated lncRNAs across chromosomes. (**K**) Overlapping distribution of dysregulated mRNAs and lncRNAs, indicating transcriptome reshaping during ferroptosis. NC: negative control (treated with DMSO). *: *p* < 0.05, **: *p* < 0.01.

To investigate transcriptional changes in glioma cells post-ferroptosis, we used the recognized ferroptosis inducer erastin to trigger ferroptosis in U87 glioma cells. We used RNA sequencing to compare erastin-treated and control groups, revealing that 20 µM erastin was more effective than 10 µM in inducing ferroptosis in glioma cells, showing statistical significance compared to the control. Erastin treatment significantly induced ferroptosis in cells, while the negative control group maintained normal cellular morphology. This suggests that Erastin at a concentration of 20 μM effectively induces ferroptosis, supporting its role as a ferroptosis inducer (Figure 1A). Moreover, the expression of the ferroptosis marker gene *GPX4* was significantly reduced following erastin treatment (Figure 1B), confirming the occurrence of ferroptosis.

### 3.2. Transcriptional Changes After Ferroptosis in Glioma Cells

Following rigorous quality control of the raw RNA sequencing data, we identified 149,802 mRNAs, 171,777 known lncRNAs, and 5203 novel predicted lncRNAs from the three erastin-induced U87 glioma cell samples and their paired DMSO treated controls. The novel lncRNAs were characterized as non-coding transcripts using CPC, CNCI, and PFAM tools (Figure 1C). Compared to the controls, 1312 mRNAs and 550 lncRNAs were significantly upregulated, while 1295 mRNAs and 537 lncRNAs were significantly downregulated in ferroptosis glioma cells [|log2FC| ≥ 1, *p*-value ≤ 0.05, and mean expression value of two-thirds of the samples in case/control group ≥1] (Figure 1D). Volcano plots (Figure 1E,F) and cluster analysis (Figure 1G,H) illustrate the distribution and expression patterns of differentially expressed mRNAs and lncRNAs between ferroptotic glioma samples and the control group. The differential expression levels of mRNAs and lncRNAs distinctly differentiated ferroptotic samples from normal control samples. The top five significantly dysregulated mRNAs and lncRNAs and their functions are listed in Table S1. These dysregulated mRNAs and lncRNAs were distributed throughout the genome, with overlaps observed across most chromosomes (Figure 1I–K). These findings suggest that the transcriptome profile during ferroptosis undergoes comprehensive reshaping, potentially driven by dynamic interactions between coding mRNAs and long non-coding RNAs.

### 3.3. Various Predictions of lncRNA Target Genes and TFs

We further studied lncRNAs, which exert their biological functions through various mechanisms, including direct base pairing with mRNAs, cis-regulation, trans-regulation, and ceRNA regulation, by sequestering microRNAs to affect downstream mRNA expression.

### 3.4. lncRNA Target Prediction via Base Pairing

**Figure 2:**
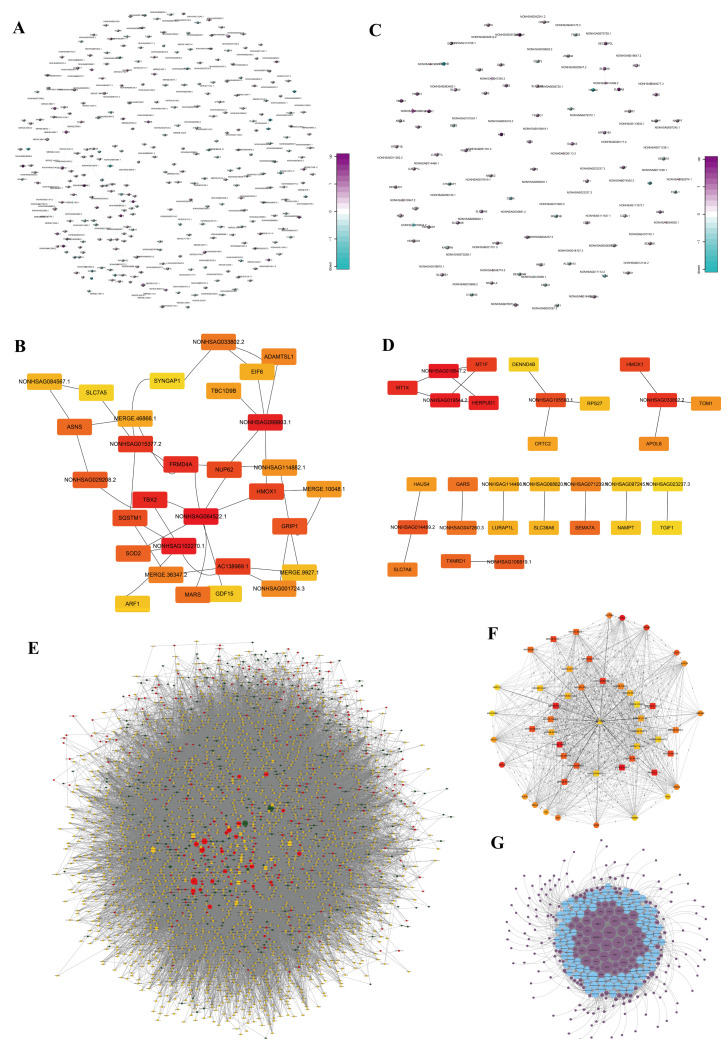
Predictions of lncRNA target genes and TFs: (**A**) Network of 474 nodes (191 lncRNAs and 283 mRNAs) and 365 connections predicted via LncTAR based on RNA pairings. (**B**) Hub model with NONHSAG064522.1 targeting 16 mRNAs, including AC138969.1, *TBX2*, *FRMD4A*, *HMOX1*, *and NUP62*. (**C**) Cis-regulation network of 75 lncRNAs and 63 mRNAs with 80 connections based on co-expression and position analysis. (**D**) Core network highlighting the NONHSAG019547.2/NONHSAG019544.2-*MT1F*/*MT1X*/*HERPUD1* and NONHSAG033802.2-*HMOX1*/*TOM1*/*APOL6* axes. (**E**) ceRNA network with 200 lncRNAs, 206 microRNAs, 322 mRNAs, and 48,025 connections. (**F**) Hub module with miR-186-3p and *SLC7A11* as key regulators, showing strong expression correlation. (**G**) TF–lncRNA network constructed using JASPAR and TFBS Tools, involving 100 lncRNAs and 237 TFs.

We employed lncTAR to predict the target genes of differentially expressed lncRNAs based on the minimum free energy of RNA molecule pairings. This analysis yielded 474 network nodes comprising 191 lncRNAs and 283 mRNAs and allowed for the construction of a network with 365 connecting bridges (Figure 2A). In this network, a single lncRNA was correlated with up to 16 coding genes, and one coding gene was targeted by up to 11 lncRNAs. The hub model was identified using Cytoscape, with NONHSAG064522.1 targeting 16 coding mRNAs and significant pairings, such as *AC138969.1*, *T-Box Transcription Factor 2* (*TBX2*), *FERM Domain Containing 4A* (*FRMD4A*), *Heme Oxygenase 1* (*HMOX1*), and *Nucleoporin 62* (*NUP62*) (Figure 2B).

### 3.5. lncRNA Target Prediction via Cis-Regulation

Regulation of mRNAs by lncRNAs can occur through cis or trans modes. This prediction is based on the co-expression of lncRNAs and mRNAs, and relative position analysis helps determine the existence of cis-targeted regulation. In this study, a network of 75 lncRNAs and 63 mRNAs connected by 80 edges was established (Figure 2C). The core network highlighted crucial control axes, such as NONHSAG019547.2/NONHSAG019544.2-*Metallothionein 1F* (*MT1F*)/*Metallothionein 1X* (*MT1X*)/*Homocysteine Inducible ER Protein With Ubiquitin Like Domain 1* (*HERPUD1*) and NONHSAG033802.2-*HMOX1*/*Target Of Myb1 Membrane Trafficking Protein* (*TOM1*)/*Apolipoprotein L6* (*APOL6*) (Figure 2D).

### 3.6. Prediction of ceRNA Networks and Hub Module Selection

We predicted miRNAs complementary to the differentially expressed lncRNAs and identified miRNAs that target mRNAs. Using miRNAs as bridges, we constructed a lncRNA–miRNA–mRNA ceRNA network. Subsequently, a network comprising 200 lncRNAs, 206 miRNAs, and 322 mRNAs was established with 48,025 connecting edges (Figure 2E). Specific regulatory axes within this network had significant roles, particularly the hub module involving miR-186-3p and *SLC7A11* (Figure 2F). The correlation between mRNA and lncRNA expression was set at >0.95, with a correlation coefficient of <0.01.

Analysis of these three lncRNA target mRNA regulation methods revealed varied results. However, the existence and functional role of these regulatory axes in ferroptosis requires further validation at the cellular level.

### 3.7. Transcription Factor Analysis

Transcription factors bind to DNA to regulate transcription in a sequence-specific manner. They are crucial in regulating gene expression by specifically binding to gene regulatory regions. They can also bind to lncRNA promoter regions, thereby influencing lncRNA expression and cancer pathogenesis [1]. Using the JASPAR database and TFBS Tools, we predicted TFs from the binding sites within 2000 bp upstream and 500 bp downstream of the lncRNA start sites. We focused on the top 100 lncRNAs in the co-expression network and their predicted paired 237 TFs to construct the network (Figure 2G). Among these, NONHSAT168932.1 matched 62 TFs, including Zinc Finger Protein 354C (ZNF354C), Myeloid Zinc Finger 1(MZF1), Forkhead Box L1 (FOXL1), and Pancreatic And Duodenal Homeobox 1 (PDX1). NONHSAT047040.2 was associated with 54 TFs, including ZNF354C, Rhox Homeobox Family Member 1 (RHOXF1), MZF1, FOXL1, and PDX1, which belong to different factor families, such as the C_2_H_2_-type zinc finger, Homeodomain, and fork head/winged helix families. Other lncRNAs, such as NONHSAT028840.2, NONHSAT226766.1, NONHSAT242831.1, and NONHSAT177508.1, were associated with more than 45 TFs. ZNF354C, RHOXF1, MZF1, FOXL1, and PDX1 were correlated with more than 80 lncRNAs, with ZNF354C having the strongest association with 95 lncRNAs.

### 3.8. GO, Pathway, and Disease Functional Enrichment Analyses

The objective of GO enrichment analysis was to delineate the functional performance of DEGs in the experimental samples. Pathway enrichment analysis has revealed the coordination of biological functions among different genes, thus enhancing our understanding of gene biology. Additionally, the disease enrichment of target gene sets provides insights into the related diseases in which these differential genes are enriched. For our analysis, mRNAs targeted by the lncRNAs were used as target gene sets.

**Figure 3:**
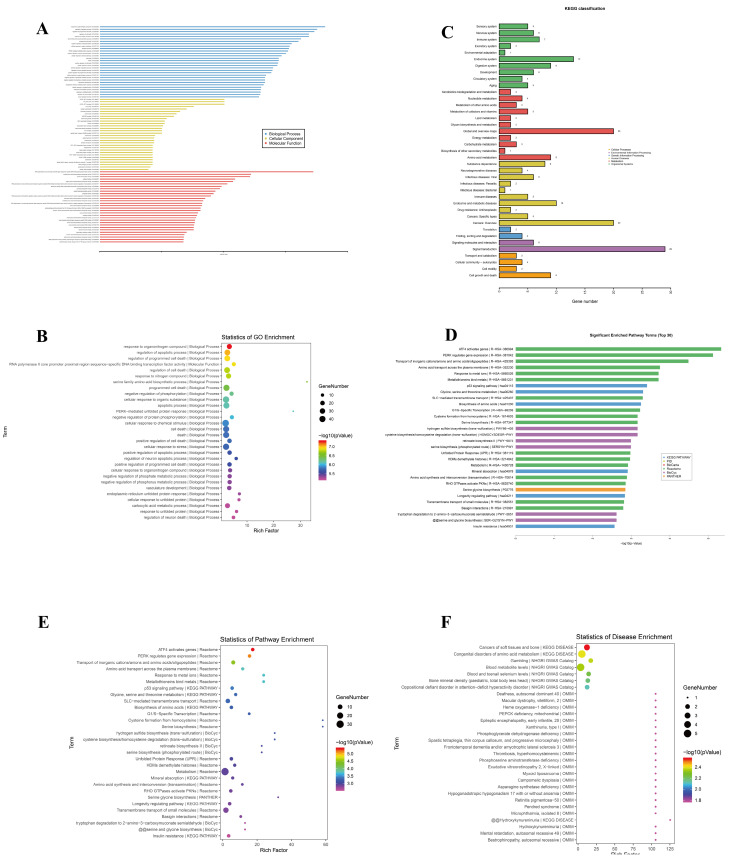
GO and pathway enrichment analyses of target gene sets: (**A**) Top 30 enriched GO terms for dysregulated lncRNAs in terms of cellular components (CCs), molecular functions (MFs), and biological processes (BPs). (**B**) Top 30 enriched GO terms overall, with significant terms including response to organonitrogen compounds, regulation of apoptotic processes, and TF activity. (**C**) KEGG pathway classification shows 234 genes in 39 categories, mainly related to cancer and metabolism. Top categories include signal transduction and endocrine and metabolic diseases. (**D**) Top 30 enrichment pathways from various databases, including Reactome, KEGG PATHWAY, BioCyc, and PANTHER, with highlighted pathways such as ATF4 activation and p53 signaling. (**E**) Specific enriched KEGG pathways, emphasizing cancer and metabolic pathways. (**F**) Disease enrichment analysis, with the top disease category being cancers of soft tissues and bone, focusing on cancer and metabolic-related diseases.

GO categorizes the characteristics of genes and gene products into three domains: cellular components (CCs), molecular functions (MFs), and biological processes (BPs). The top 30 enriched GO terms for the dysregulated lncRNAs in each category are presented in Figure 3A, while the top 30 GO terms across all significant GO items are displayed in Figure 3B. The most significant terms included responses to organonitrogen compounds, regulation of apoptotic processes, and RNA polymerase II core promoter proximal region sequence-specific DNA binding TF activity in CC; DNA Damage Inducible Transcript 3 (DDIT3)-ATF3 complex and Lewy body core in MF; and neutral amino acid transmembrane transporter activity and protein dimerization activity in BP. The greatest enrichment among all significant GO items was the response to organonitrogen compounds (Figure 3B), likely due to erastin (an organonitrogen compound) treatment. Moreover, GO functional enrichment analysis indicated that the functions of differentially expressed lncRNAs were predominant in various cell death types, gene expression regulation processes, and central nervous system diseases.

Next, KEGG pathway classification was obtained according to the different classes of genes. A total of 234 genes correlated with 39 differential classifications in the KEGG pathway classifications, primarily related to cancer and metabolism (Figure 3C). The top five classifications comprised signal transduction, global and overview maps, cancer, endocrine system, and endocrine and metabolic diseases. The top 30 enriched pathways are shown in Figure 3D,E, with the most enriched pathways in each database including ATF4 activation genes (Reactome), the p53 signaling pathway (KEGG PATHWAY), cysteine biosynthesis/homocysteine degradation (BioCyc), and serine glycine biosynthesis (PANTHER).

Disease enrichment analysis of target gene sets aids in understanding diseases associated with DEGs. This analysis helps pinpoint potential diseases affected by altered gene expression and provides a pathway for understanding the broader implications of these changes in clinical settings. In this study, the most enriched diseases were cancers of the soft tissues and bone, with most of the enriched diseases being cancer and metabolism-related diseases (Figure 3F).

### 3.9. Differential Gene–Protein Interaction Network, Co-Expression, and lncRNA–mRNA–RBP Prediction Analyses

#### 3.9.1. Differential Gene–Protein Interaction Network Analysis

**Figure 4:**
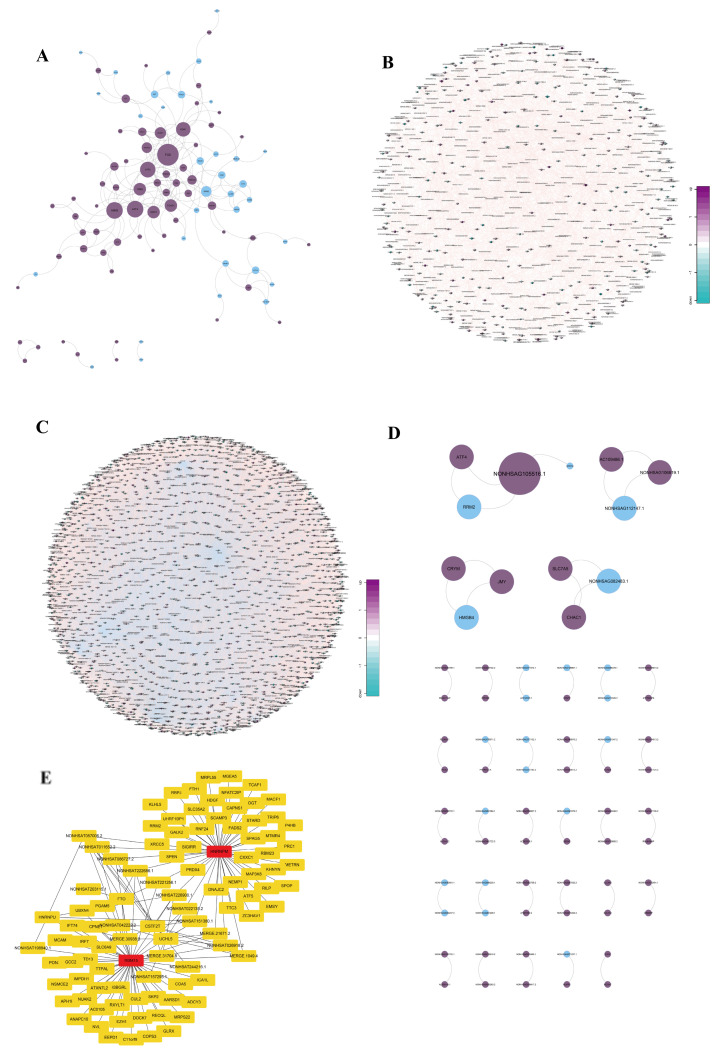
Differential gene interaction and co-expression analysis: (**A**) PPI network of differentially expressed mRNAs using STRING and Cytoscape, with 97 nodes and 185 edges, highlighting key interactions such as FOS-EGR1 and ATF4-DDIT3. (**B**) Co-expression network based on differentially expressed lncRNAs and mRNAs using Pearson correlation, with 778 genes and 1219 edges. (**C**) Filtered co-expression network, with interactions having an absolute correlation ≥ 0.99 and *p*-value < 0.05, featuring 41 lncRNAs and 30 mRNAs. (**D**) lncRNA–mRNA–RBP network derived from ENCODE predictions, showing 489 lncRNAs, 1470 mRNAs, and 38 common RBPs. (**E**) Hub modules of RBPs binding to multiple lncRNAs.

Proteins, which are fundamental to life and various biological activities, form PPI networks that are crucial for biological signaling, gene regulation, metabolic processes, and cell cycle control. We analyzed the differentially expressed mRNAs for protein–protein interactions using the STRING database. Using Cytoscape with protein–protein interaction scores from STRING, we constructed the final network. The network comprised 97 nodes and 185 edges, with a cut-off score greater than 400 (Figure 4A). Hub PPIs such as Fos Proto-Oncogene (FOS)-Early Growth Response 1 (EGR1)/Dual Specificity Phosphatase 1 (DUSP1)/HMOX1, Asparagine Synthetase (ASNS)-ATF4, ATF4-DDIT3/ATF3/Homocysteine Inducible ER Protein With Ubiquitin Like Domain 1 (HERPUD1), ATF3-DDIT3, and FOXO1-PPARG Coactivator 1 Alpha (PPARGC1A), were filtered based on gene edges greater than 10 and scores greater than 920.

#### 3.9.2. Differential Gene Co-Expression Analysis

Co-expression analysis allows for the prediction of gene relationships through the comparison of expression trends across different samples. We calculated the correlation coefficients and *p*-values between DEGs using their expression levels and the Pearson model, resulting in a co-expression relationship. A co-expression network was then constructed based on variations in gene expression signals, revealing the regulatory relationships and directions between the genes. Such networks allow researchers to identify core regulatory genes that change during experiments. In this study, the co-expression network, based on differentially expressed lncRNAs and mRNA expression-normalized data using the Pearson algorithm and *p*-value, consisted of 778 genes connected by 1219 edges (Figure 4B). Interactions with an absolute correlation coefficient value ≥ 0.99 and *p*-value < 0.05 were filtered out (Figure 4C), including 41 lncRNAs and 30 mRNAs connected by 42 bridges. However, the significance of NONHSAG105516.1-*ATF4*/*Ribonucleotide Reductase Regulatory Subunit M2* (*RRM2*) in this network requires further investigation.

#### 3.9.3. lncRNA–mRNA–RBP Prediction Analysis

We sourced RBPs from the ENCODE database and analyzed them based on lncRNA–mRNA correlation results (correlation > 0.95) to predict RBPs that commonly bind to both lncRNA and mRNA. Triangles in figures represent lncRNAs, circles represent mRNAs, and stars represent RBPs, including the hub modules. As RBPs bind to lncRNAs or mRNA, they take on crucial roles in post-transcriptional processes such as splicing, modification, transport, cellular localization, stability, translation, and degradation, thus regulating RNA metabolism. Based on the 24,484 lncRNA–mRNA–RBP connections predicted by ENCODE, 489 lncRNAs and 1470 mRNAs were found to share 38 common RBPs in the network (Figure 4D). Subsequently, hub modules were filtered using Cytoscape. RBPs, such as RNA Binding Motif Protein 15 (RBM15) and Heterogeneous Nuclear Ribonucleoprotein M (HNRNPM), could bind to multiple lncRNAs, such as MERGE.1049.4, NONHSAT057006.2, and others, thereby regulating mRNA expression (Figure 4E).

### 3.10. Interplay Between Ferroptosis and Endoplasmic Reticulum Stress

Increasing evidence indicates a complex interplay between the regulatory mechanisms of ferroptosis and endoplasmic reticulum (ER) stress. Understanding the interaction between ferroptosis and ER stress is crucial for elucidating the mechanisms underlying cell death and developing innovative therapies. In this RNA-seq study, we observed that, following the induction of ferroptosis in glioma cells, several genes involved in ER stress exhibited significant differential expression. We compiled a gene set related to ER stress from “GeneCard” and “MSigDB” and identified 1688 genes (Table S2), of which 254 showed differential expression post-ferroptosis (Table S3). The top 10 genes with increased expression were *Transferrin Receptor* (*TFRC*), *S-Phase Kinase Associated Protein 1* (*SKP1*), *Cyclin Dependent Kinase Inhibitor 3* (*CDKN3*), *Beta-Transducin Repeat Containing E3 Ubiquitin Protein Ligase* (*BTRC*), *SEC61 Translocon Subunit Beta* (*SEC61B*), *BAG Cochaperone 6* (*BAG6*), *Catenin Beta 1* (*CTNNB1*), *CIRBP*, *Basigin* (*BSG*), *and BSCL2 Lipid Droplet Biogenesis Associated* (*BSCL2*). Conversely, the top 10 genes with decreased expression were *Fas Cell Surface Death Receptor* (*FAS*), *CDC28 Protein Kinase Regulatory Subunit 1B* (*CKS1B*), *Baculoviral IAP Repeat Containing 2* (*BIRC2*), *Lamin B Receptor* (*LBR*), *Succinate Dehydrogenase Complex Flavoprotein Subunit A* (*SDHA*), *Ribosomal Protein Lateral Stalk Subunit P0* (*RPLP0*), *YY1 Transcription Factor* (*YY1*), *Exosome Component 10* (*EXOSC10*), *DnaJ Heat Shock Protein Family* (*Hsp40*) *Member C10* (*DNAJC10*), *and Fukutin Related Protein* (*FKRP*). The specific roles of these genes in glioma ferroptosis and ER stress remain unclear and warrant further investigation.

### 3.11. Role of CIRBP in Glioma Ferroptosis

**Figure 5:**
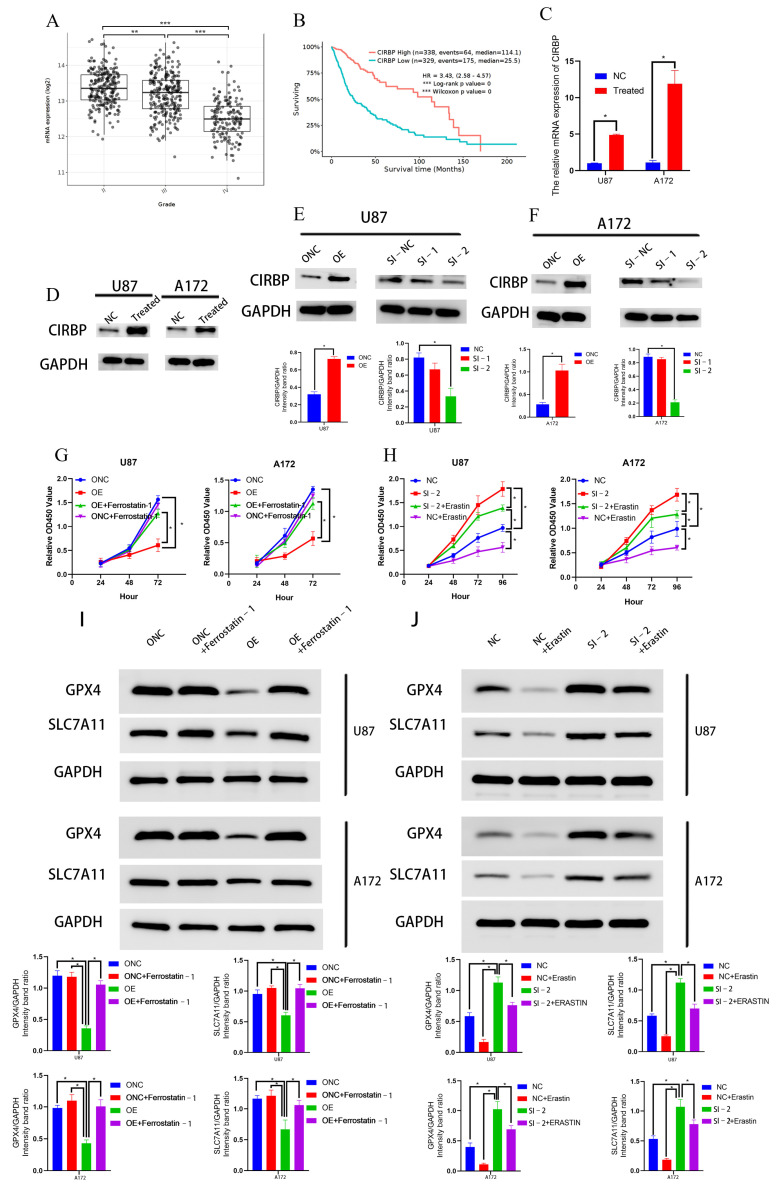
Role of CIRBP in glioma and ferroptosis: (**A**) CIRBP expression decreases with higher glioma grades (TCGA data). (The data presented in this figure are derived from the TCGA database, which does not include RNA-seq data for normal brain tissue). (**B**) Lower CIRBP expression correlates with poorer prognosis in patients with glioma. (**C**) Increase in CIRBP expression (~4.9-fold) in U87 and (~10.8-fold) in A172 cells after ferroptosis induction via qRT-PCR. (**D**) Western blots showing increased CIRBP protein levels in U87 and A172 cells after ferroptosis. (**E**,**F**) Verification of CIRBP overexpression and knockdown efficiency in U87 and A172 cells. (**G**,**H**) CIRBP overexpression or knockdown inhibits or promoted glioma cell proliferation (CCK8 assay), which was reversible after treatment with a ferroptosis inhibitor or a ferroptosis inducer, respectively. (**I**,**J**) Expression of GPX4 and SLC7A11 after CIRBP overexpression with ferroptosis inhibitor and knockdown with a ferroptosis inducer at the protein level. OE: overexpression; ONC: negative control of overexpression; SI-1: siRNA-1; SI-2: siRNA-2; NC: negative control of siRNA. *: *p* < 0.05, **: *p* < 0.01, ***:*p* < 0.001.

Although CIRBP is associated with ferroptosis, its role in glioma has not yet been extensively studied. By analyzing glioma data from The Cancer Genome Atlas (TCGA) database, we found that the expression of *CIRBP* decreased as the grade of glioma increased (Figure 5A), and patients with low *CIRBP* expression tended to have a poorer prognosis than those with high expression (Figure 5B), suggesting that CIRBP may inhibit the progression of glioma. RNA-seq results indicated that after inducing ferroptosis in U87 cells, *CIRBP* expression increased approximately 9.0-fold. Validation by qRT-PCR showed an increase of about 4.9-fold in U87 and about 10.8-fold in A172 cells in *CIRBP* expression following ferroptosis in U87 and A172 cells (Figure 5C). Additionally, the Western blotting analyses (Figure 5D) indicated an increase in CIRBP protein levels after ferroptosis in these cells. To assess the role of CIRBP in ferroptosis, we analyzed its effect on erastin-induced and ferrostatin 1-inhibited ferroptosis in glioma cells and expression of GPX4 and SLC7A11, which are markers of ferroptosis.

The efficiency of CIRBP overexpression and knockdown in U87 and A172 cells was verified by successful knockdown (Figure 5E,F).

The results of the CCK8 assay suggested that the overexpression of CIRBP inhibited the proliferation of glioma cells, and this inhibitory effect could be partially reversed using a ferroptosis inhibitor ferrostatin 1 (Figure 5G). Inversely, CIRBP knockout in glioma cells promoted glioma cell proliferation and reduced their sensitivity to erastin-induced ferroptosis (Figure 5H).

Additionally, Western blot results showed that overexpression of CIRBP led to the downregulation of GPX4 and SLC7A11 and could been reversed by ferroptosis inhibitor ferrostatin 1 (Figure 5I). In contrast, knocking out CIRBP resulted in increased expression of these proteins and could been reversed by ferroptosis inducer erastin (Figure 5J). These findings suggest that by promoting ferroptotic death of glioma cells, CIRBP is essential to glioma ferroptosis. However, the specific mechanisms by which CIRBP influences ferroptosis and glioma progression warrant further research to comprehensively elucidate its therapeutic potential.

## 4. Discussion

Gliomas, the most prevalent intracranial malignant tumors, are known for their poor prognosis and originate from neuroepithelial cells [1,2]. Despite advancements in basic research and novel therapeutic strategies, patient prognosis remains poor [7]. Ferroptosis, a recently identified form of programmed cell death, is still being explored in the context of gliomas. The exact role of ferroptosis in glioma pathogenesis and its potential as a novel therapeutic target warrant further investigation. Current research on glioma-associated ferroptosis primarily involves bioinformatic analysis, comparing ferroptosis gene sets from published studies across various cancer types using glioma RNA sequencing and corresponding clinical data from open databases. However, these studies are limited by the inconsistency of ferroptosis-related genes across different tumor types and the potential for inaccuracies when applying findings from other tumors to gliomas. To address these limitations, we investigated the differential expression of lncRNAs and mRNAs in gliomas. Using various analytical methods, we aimed to elucidate the potential relationships between ferroptosis and gliomas, as well as the mechanisms underlying these relationships, paving the way for future research and potential therapeutic interventions.

Using next-generation RNA sequencing analysis, we demonstrated significant differences in the expression of mRNAs and lncRNAs associated with ferroptosis in gliomas. We identified 149,802 mRNAs, 171,777 lncRNAs, and 5203 novel lncRNAs. Notably, 2607 mRNAs (1312 upregulated and 1295 downregulated) and 1087 lncRNAs (550 upregulated and 537 downregulated) were significantly differentially expressed in erastin-treated U87 glioma cells compared to untreated cells. We acknowledge that the RNA sequencing data in this study were derived solely from the U87 cell line, which may introduce cell line-specific bias and limit the generalizability of the results to other GBM cell lines or primary tumor samples. Although the U87 cell line is one of the most widely used models in GBM research, significant differences in gene expression may exist between different GBM cell lines. Therefore, our findings require further validation in other GBM models. Future studies will include a variety of GBM cell lines and primary tumor samples to enhance the general applicability of our findings and further elucidate the molecular mechanisms of Erastin’s effects. Further analysis was conducted to elucidate the functions of the deregulated lncRNAs through GO and pathway analyses. These lncRNAs are predominantly associated with various types of cell death, gene expression regulation processes, central nervous system diseases, and cancers. Intriguingly, pathways such as Lewy body core, p53 signaling, cysteine biosynthesis/homocysteine degradation, and serine/glycine biosynthesis, which are linked to protein ubiquitination, were identified. Ubiquitination, a critical post-translational modification in the proteasomal degradation system, acts in both normal physiological states and disease conditions. Lewy bodies, which are the hallmarks of Parkinson’s disease, are highly ubiquitinated. This is influenced by K63-linked ubiquitination of alpha-synuclein and synphilin-1 [29]. The tumor suppressor gene p53 promotes its own polyubiquitination by enhancing the HDM2–HDMX interaction [30,31]. In addition to other types of ubiquitination, non-lysine residues such as serine and cysteine have important roles in the ubiquitination process [32]. Our results suggest that lncRNA targets were enriched in various ubiquitination-related pathways. However, the exact role of ubiquitination in glioma ferroptosis and its underlying mechanisms require further investigation.

In our experiments, we observed significant changes in the expression of numerous mRNAs and lncRNAs in U87 glioma cells following erastin-induced ferroptosis. These findings share some commonalities with other ferroptosis-related studies, such as those by Zhuo et al. [33] and Liu et al. [34], while also exhibiting certain unique characteristics.

Our study shows that erastin treatment significantly downregulated the expression of GPX4 and SLC7A11, confirming the occurrence of ferroptosis in glioma cells. This is consistent with the findings of Zhuo et al., who also demonstrated the critical roles of GPX4 and the system Xc^−^ in ferroptosis [33]. In their study, they constructed a ferroptosis-related risk signature based on 25 genes using TCGA and CGGA datasets, further highlighting the importance of ferroptosis-related genes in predicting the prognosis of glioma patients. Similarly, our RNA-seq data revealed significant changes in the expression of multiple ferroptosis-related genes during erastin-induced ferroptosis, further supporting the critical role of these genes in the regulation of ferroptosis.

Additionally, Liu et al. analyzed the expression profiles of ferroptosis-related genes in glioma using multi-omics data and identified 19 genes that were highly associated with glioma progression [34]. Most of these genes were involved in lipid peroxidation, iron metabolism, and oxidative stress regulation, which aligns with the transcriptional remodeling observed in our experiments. In particular, we found that the upregulated and downregulated lncRNAs and mRNAs were involved in several ferroptosis-related signaling pathways, such as the p53 signaling pathway, cystine synthesis, and homocysteine metabolism pathways, all of which were also identified as key ferroptosis pathways in Liu’s study.

While there are commonalities across these studies, the uniqueness of our research lies in the identification of a large number of newly predicted lncRNAs. Further bioinformatics analysis confirmed these lncRNAs as non-coding transcripts that may play crucial roles in ferroptosis regulation. This finding is consistent with the results of Huang et al. [35], who identified a ferroptosis-related lncRNA (FRL) risk model and demonstrated the important role of lncRNAs in ferroptosis and glioma prognosis. Overall, our experimental results are in line with the existing ferroptosis-related datasets in the literature while also offering new insights, particularly regarding the potential regulatory role of lncRNAs. Future research could further explore the specific mechanisms of these regulatory axes and uncover their potential in ferroptosis and glioma therapy.

Our integrated, multifaceted analysis highlights the intricate potential regulatory mechanisms of lncRNAs and mRNAs in glioma ferroptosis. We employed strategies such as base pairing, cis-regulation prediction, ceRNA analysis, gene co-expression, and lncRNA–mRNA–RBP prediction. These approaches allowed us to identify pivotal lncRNA–mRNA regulatory pairs, including NONHSAG064522.1, *TBX2*, *FRMD4A*, *HMOX1*, *NUP62;* NONHSAG019547.2, NONHSAG019544.2-*MT1F*/*MT1X*/*HERPUD1*, NONHSAG033802.2, *HMOX1*/*TOM1*/*APOL6*, NONHSAG105516.1, and *ATF4*/*RRM2*.

However, many of the filtered lncRNAs have not yet been extensively studied or reported in humans. We identified several lncRNAs targets that were differentially expressed in ferroptotic glioma cells, including *TBX2*, *FRMD4A*, *NUP62*, *TOM1*, *APOL6*, *DUSP1*, *ASNS*, *DDIT3*, *PPARGC1A*, and *FOXO1*. These genes, which have not previously been studied in the context of ferroptosis, present new avenues of exploration. In particular, *HMOX1*, which was upregulated 28 times in ferroptotic glioma cells in our study, is known to play a dual role in cancers. Although its antioxidant activity provides a protective function, its overactivation enhances ferroptosis, particularly when the ferritin buffering capacity is insufficient, leading to Fenton-mediated decomposition [36]. Additionally, HMOX1 upregulation promotes ferroptosis, and HMOX1 knockdown decreases the Fe^2+^ and reactive oxygen species (ROS) levels and suppresses lipid peroxidation in diabetic atherosclerosis [37]. Moreover, HMOX1 overexpression induces ferroptosis by suppressing GPX4 expression in osteosarcoma cells [38]. In gliomas, a paradoxical phenomenon occurs in which HMOX1 is overexpressed and correlated with worse survival [39], promoting glioma cell proliferation [40]. It is also highly expressed in macrophages, influencing T cell proliferation and iron metabolism in the glioma microenvironment [41].

The role of metallothionein genes, such as *MT1F* and *MT1X*, which are involved in the metabolism of divalent heavy metal ions and are associated with detoxification, oxidative stress, and carcinogenesis, remains unexplored in ferroptosis [42]. HERPUD1’s involvement in ferroptosis has been demonstrated in various cancers [43,44], suggesting a broad role in this cell death mechanism. *RRM2*, another gene of interest, is linked to macrophage polarization and lung cancer cell proliferation, and its inhibition induces ferroptosis in lung adenocarcinoma [45]. Additionally, the small molecule DMOCPTL, derived from a natural parthenolide, binds directly to GPX4, inducing its ubiquitination and leading to ferroptosis in breast cancer cells [46]. This highlights the potential crosstalk between apoptosis and ferroptosis and the importance of gene ubiquitination in these processes. Similarly, ATF3, a common stress sensor, has been shown to inhibit SLC7A11 expression, leading to system Xc suppression, GSH deficiency, lipid peroxidation, and, consequently, ferroptosis [47,48]. Its role in promoting ferroptosis in glioma cells via H_2_O_2_ and Fe^2+^ and the potential crosstalk between ferroptosis and apoptosis require further elucidation [14]. Our study underscores the complex regulatory landscape of lncRNA–mRNA interactions in glioma ferroptosis. The identified regulatory pairs and diverse roles of various genes present a rich tapestry of molecular interactions and pathways that justify further investigation to understand their roles in glioma pathology and potential therapeutic interventions.

Furthermore, the construction of a ceRNA network advances our understanding of the ceRNA hypothesis, suggesting that lncRNAs act as molecular sponges to sequester miRNAs, modulating the availability of miRNAs to target mRNAs. The central roles of miR-186-3p and *SLC7A11* within the hub module of the network underscore their potential as key regulators of gene expression mechanisms, providing deeper insights into their roles in disease processes and identifying novel targets for therapeutic intervention. SLC7A11 is a key component of the system Xc⁻ transporter, responsible for importing extracellular cystine and exporting intracellular glutamate, a process that is crucial for the synthesis of glutathione (GSH). GSH, as the primary intracellular antioxidant, protects cells from damage caused by reactive oxygen species (ROS) and lipid peroxidation, thereby inhibiting ferroptosis. The upregulation of SLC7A11 enhances GSH production, strengthening the cell’s resistance to oxidative stress and preventing ferroptosis. Conversely, the loss or inhibition of SLC7A11 leads to GSH depletion, accumulation of lipid peroxides, and, ultimately, the promotion of ferroptosis [49].

miR-186, a key microRNA, has been shown to have regulatory effects in various cancers [50]. Based on the prediction of miR-186 downstream target genes, SLC7A11 is identified as one of its target genes. However, there are currently no studies reporting that miR-186 directly downregulates SLC7A11 expression to inhibit the function of the system Xc⁻. This potential downregulation could weaken the cell’s antioxidant defense system, leading to a decrease in GSH levels and the accumulation of ROS, ultimately accelerating the process of ferroptosis. Therefore, miR-186 not only potentially regulates the cell’s antioxidant capacity through direct regulation of SLC7A11 but also indirectly influences lipid metabolism and oxidative stress responses, making it an important modulator of ferroptosis.

In summary, SLC7A11 and miR-186 may play complementary roles in the regulation of ferroptosis. SLC7A11 protects cells from ferroptosis by maintaining GSH levels, while miR-186 promotes ferroptosis by suppressing SLC7A11 expression. These findings underscore their roles as critical regulators in gene expression mechanisms and highlight their potential as therapeutic targets in ferroptosis-related diseases. Further investigation into the molecular mechanisms of SLC7A11 and miR-186 is essential for understanding the complex regulatory network of ferroptosis.

During our investigation of ferroptosis-induced differential mRNA expression in U87 cells, we observed concurrent alterations in several genes related to ER stress; ER stress is a cellular response to various stressors that leads to abnormal protein folding and activates a series of adaptive signaling pathways. These pathways maintain cellular homeostasis by regulating protein folding, protein degradation, and various cell death processes. Prolonged or excessive ER stress results in cellular dysfunction and death. While the occurrence of ferroptosis induces ER stress by affecting iron metabolism and lipid peroxidation, ER stress regulates the onset of ferroptosis by influencing antioxidant responses and lipid metabolism.

The endoplasmic reticulum (ER) plays a critical role in the initiation of ferroptosis by regulating lipid metabolism [51]. The ER is involved in the synthesis, folding, modification, and transport of cellular lipids. When ER stress occurs, these metabolic processes are disrupted, leading to the accumulation of lipid peroxides, which is a hallmark of ferroptosis.

Firstly, ER stress activates the unfolded protein response (UPR), which aims to restore ER homeostasis. However, if the stress is too severe or prolonged, the UPR regulates multiple lipid metabolic pathways, promoting ferroptosis. For example, stress signals upregulate transcription factors such as X-box binding protein 1 (XBP1) and activating transcription factor 6 (ATF6), inducing the synthesis of polyunsaturated fatty acids (PUFAs) [52]. PUFAs are the target lipids in ferroptosis because they are prone to oxidation, forming lipid peroxides, whose accumulation is a major trigger for ferroptosis. Secondly, ER stress exacerbates lipid peroxidation by inhibiting the activity of glutathione peroxidase 4 (GPX4). GPX4 is the only known enzyme capable of reducing phospholipid hydroperoxides in cells. Inhibition of GPX4 leads to the accumulation of lipid peroxides, triggering ferroptosis. Studies suggest that ER stress may regulate GPX4 expression or indirectly reduce its activity through reactive oxygen species (ROS) signaling, further accelerating lipid peroxidation [51]. Additionally, interactions between the ER, mitochondria [53], and lysosomes [54] may also play key roles in regulating ferroptosis. The ER modulates the distribution of intracellular iron and the production of lipids through its metabolic coupling with these organelles, which is crucial for ferroptosis. ER stress acts as a central regulator in these processes, ultimately initiating ferroptosis by modulating lipid metabolism and iron homeostasis. Specifically, ER stress may contribute to ferroptosis by influencing lipid metabolism and oxidative stress responses. When the UPR is activated, it can induce redox imbalance by upregulating certain genes (such as ATF4 and CHOP), leading to the accumulation of lipid peroxides, which are closely linked to the onset of ferroptosis [55]. Additionally, the UPR can regulate iron metabolism-related genes (such as HO-1) to increase intracellular iron levels, further driving ferroptosis [51]. Moreover, activation of ER stress pathways such as PERK and IRE1 has been found to directly or indirectly affect the expression and activity of factors related to ferroptosis. In gliomas, the potential interplay and regulatory relationship between ferroptosis and ER stress remain unclear, and the specific mechanisms involved in these processes require further in-depth studies.

When discussing ER stress, it is important to connect it to ferroptosis and other forms of cell death, such as apoptosis, as they may share similar endpoints—cell death induced by stress. Although ferroptosis and apoptosis are morphologically distinct, both involve cellular responses to endogenous or exogenous stress that trigger cell death. In the case of ER stress, the accumulation of unfolded or misfolded proteins in the ER activates the UPR, ultimately leading to cell fate decisions. There are also intersections between apoptosis and ferroptosis. For example, the p53 signaling pathway plays a crucial role in both forms of cell death. Under conditions of ER stress, p53 activation can induce apoptosis by regulating genes such as BAX and PUMA [56]. At the same time, p53 can enhance cellular sensitivity to ferroptosis by downregulating SLC7A11, a key player in glutathione synthesis, thereby facilitating the ferroptotic process [57]. Thus, the similarities between ER stress and ferroptosis lie in their regulation of cell death through shared stress response pathways, such as UPR and p53, as well as in promoting lipid peroxidation through redox imbalance and iron metabolism, which parallels the oxidative stress-induced cell death mechanisms seen in apoptosis.

In this study, we focused on CIRBP and conducted preliminary research on its role in glioma ferroptosis. Cold-inducible RNA binding protein modulates target mRNAs at post-transcriptional level expression, and it is induced by cold shock, ROS, and hypoxia [58]. It acts as a tumor suppressor by binding directly to p53, increasing levels of Fe^2+^ and ROS, and inhibiting GSH expression, which induces ferroptosis in pancreatic cancer cells [59]. When analyzing the role of CIRBP, it is important to note that its interaction with p53 may play a role in regulating ferroptosis [59]. p53 is a key stress response regulator that can promote ferroptosis by downregulating SLC7A11 expression, thereby increasing intracellular iron levels, which leads to the accumulation of lipid peroxides and the onset of ferroptosis [57]. CIRBP may enhance p53’s regulatory effects on downstream genes, thus influencing iron metabolism and lipid metabolism.

Additionally, the impact of CIRBP on iron levels warrants further investigation. Overexpression of CIRBP may increase intracellular iron levels by regulating iron transport-related genes, thereby accelerating ferroptosis [60].

Following treatment with erastin, CIRBP expression increases, and its knockdown inhibits erastin-induced ferroptosis by ferritinophagy during renal ischemia-reperfusion injury [60]. In intracranial tumors, CIRBP is overexpressed in tumor-adjacent areas compared to its expression in metastatic lesions in the brain. This overexpression has been linked to the invasive growth of brain metastases and is a predictive biomarker for the recurrence of brain metastases [61]. However, the specific role of CIRBP in glioma ferroptosis remains unclear. Our experimental results and survival curve analysis revealed that patients with glioma with high CIRBP expression had longer overall survival periods. After ferroptosis induction in gliomas, CIRBP expression increased, with notably higher expression in low-grade gliomas than in high-grade gliomas. Patients with low CIRBP expression generally have a worse prognosis than those with high CIRBP expression, suggesting that *CIRBP* may play a role as a tumor suppressor gene in gliomas. Our findings indicate that inhibiting CIRBP expression counteracts erastin-induced ferroptosis in U87 and A172 cells; however, the specific mechanisms underlying this effect require further investigation.

This study represents the first lncRNA–mRNA co-expression analysis performed using ferroptosis-induced glioma cells. By examining the co-expression of coding and non-coding genes, we gained valuable insights into ferroptosis in glioma cells. We identified differentially expressed mRNAs and lncRNAs and explored their potential relationships in glioma ferroptosis. However, further studies are required to elucidate the specific molecular mechanisms underlying this process. Our investigation is preliminary, and we have made our data available on the NCBI database to offer a comprehensive and informative dataset for researchers interested in the field of glioma ferroptosis.

## Data Availability

The dataset is available upon request from the authors.

## Supplementary Materials

The following supporting information can be downloaded at: https://www.mdpi.com/article/10.3390/biomedicines13010041/s1, Table S1: Top five significantly dysregulated mRNAs and lncRNAs and their functions; Table S2: Gene set related to ER stress from “GeneCard” and “MSigDB”; Table S3: 254 differential expressed ER stress related genes after post-ferroptosis.
